# New strategy for designing orangish-red-emitting phosphor via oxygen-vacancy-induced electronic localization

**DOI:** 10.1038/s41377-019-0126-1

**Published:** 2019-01-30

**Authors:** Yi Wei, Gongcheng Xing, Kang Liu, Guogang Li, Peipei Dang, Sisi Liang, Min Liu, Ziyong Cheng, Dayong Jin, Jun Lin

**Affiliations:** 1 Engineering Research Center of Nano-Geomaterials of Ministry of Education, Faculty of Materials Science and Chemistry, China University of Geosciences, 388 Lumo Road, 430074 Wuhan, People’s Republic of China; 2 State Key Laboratory of Rare Earth Resource Utilization, Changchun Institute of Applied Chemistry, Chinese Academy of Sciences, 130022 Changchun, People’s Republic of China; 3 Hunan Key Laboratory for Super-microstructure and Ultrafast Process, School of Physics and Electronics, Central South University, 410083 Changsha, Hunan People’s Republic of China; 4 State Key Laboratory of Powder Metallurgy, Central South University, 932 South Lushan Road, 410083 Changsha, Hunan People’s Republic of China; 5 Institute for Biomedical Materials and Devices (IBMD), Faculty of Science, University of Technology Sydney, Sydney, NSW Australia; 6 School of Applied Physics and Materials, Wuyi University, 529020 Jiangmen, Guangdong People’s Republic of China

**Keywords:** Optical materials and structures, Inorganic LEDs

## Abstract

Phosphor-converted white-light-emitting diodes (pc-WLED) have been extensively employed as solid-state lighting sources, which have a very important role in people’s daily lives. However, due to the scarcity of the red component, it is difficult to realize warm white light efficiently. Hence, red-emitting phosphors are urgently required for improving the illumination quality. In this work, we develop a novel orangish-red La_4_GeO_8_:Bi^3+^ phosphor, the emission peak of which is located at 600 nm under near-ultraviolet (n-UV) light excitation. The full width at half maximum (fwhm) is 103 nm, the internal quantum efficiency (IQE) exceeds 88%, and the external quantum efficiency (EQE) is 69%. According to Rietveld refinement analysis and density functional theory (DFT) calculations, Bi^3+^ ions randomly occupy all La sites in orthorhombic La_4_GeO_8_. Importantly, the oxygen-vacancy-induced electronic localization around the Bi^3+^ ions is the main reason for the highly efficient orangish-red luminescence. These results provide a new perspective and insight from the local electron structure for designing inorganic phosphor materials that realize the unique luminescence performance of Bi^3+^ ions.

## LEDs: Painting the town red

Phosphorescent substances that give white-light-emitting diodes a more natural reddish hue can be made by tuning the electron structure of their activator ions. Jun Lin of China’s Changchun Institute of Applied Chemistry, Chinese Academy of Sciences and colleagues combined germanium, lanthanum and bismuth oxides to manufacture a reddish phosphorescent substance that, when added to blue and green phosphors on an LED chip, produced more natural-looking light compared to conventional phosphor-converted white LEDs. LEDs are constantly being improved as an energy-efficient light source, but the colour of the light they emit can be unnaturally white or blue. Fabricating high quality red-emitting phosphors to change this has been a challenge. The researchers developed a substance with a unique reddish photoluminescence caused by electrons localizing around bismuth ions. Their approach offers a new perspective for exploring luminescence in inorganic materials.

## Introduction

Phosphor-converted white-light-emitting diodes (pc-WLED) have become the next-generation solid-state lighting source in both indoor and outdoor lighting areas^1–4^. Conventional WLEDs are composed of a blue LED chip and yellow YAG:Ce^3+^ phosphor; however, they emit cold white light because the emission spectra do not cover the red region^5^. Therefore, red phosphor is very important for producing warm white luminescence and improving the luminous efficiency. Many previous works have explored the use of red-emitting phosphor to enhance pc-WLED lighting quality. Eu^3+^-doped inorganic phosphor materials are the most frequently investigated red phosphor materials due to the typical 4*f*–4*f* partial spin and forbidden transition^6,7^. However, Eu^3+^ is rarely utilized in warm pc-WLEDs because its excitation spectra do not fit well with near-ultraviolet (n-UV) and blue light^8^. The most widely commercially available red phosphors are Eu^2+^-doped nitride phosphors^9–12^, such as CaAlSiN_3_:Eu^2+^ and Sr_2_Si_5_N_8_:Eu^2+^. Although these phosphors realize high quantum efficiency, harsh synthesis conditions (high temperature and high pressure) and deep-red emission limit their large-scale application  in the production of warm white light. In addition, Zhang et al. reported a narrow-band red-emitting SrLiAl_3_N_4_:Eu^2+^ phosphor that was obtained via facile atmospheric pressure synthesis, which could easily compensate the red component for YAG:Ce^3+^ and potentially serve as an alternative phosphor for n-UV pc-WLEDs. However, the spectral overlap still remains a large problem^13^. To date, linear red-emitting Mn^4+^-doped fluoride phosphors have attracted substantial attention, as their quantum yield can exceed 98% under blue light irradiation^14,15^. However, they have two serious drawbacks: low thermal stability and massive HF acid use. Hence, exploiting high-quality red-emitting phosphor materials remains challenging.

Recently, Bi^3+^-activated phosphors have been extensively investigated due to their unique luminescence performance^16^. Their excitation spectra are located in the n-UV area; thus, spectral overlap can be efficiently avoided. However, Bi^3+^ ions typically emit blue and green light and rarely emit red light, except in a suitable matrix, such as ScVO_4_ and ZnWO_4_^17–20^. Bi^3+^ contains a naked 6*s*6*p* energy level; hence, the surrounding coordination environment is very sensitive^21,22^. The luminescence performance of Bi^3+^ can be easily influenced by tuning the surrounding electron structure. Recently, it was demonstrated that the formation of a vacant defect could contribute to the spectral adjustment. For instance, Zhang et al.^23^ reported a giant enhancement of Bi^3+^ luminescence that was realized by generating an oxygen vacancy, which is a new strategy for exploring novel Bi^3+^-doped phosphor materials.

In this work, we develop a novel and high-quality red-emitting La_4_GeO_8_:Bi^3+^ (denoted as LGO:Bi^3+^) phosphor. Under 397 nm n-UV excitation, LGO:Bi^3+^ displays an orangish-red emission for which the peak is at 600 nm, fwhm = 103 nm, IQE = 88.3%, and EQE = 69%. Via experimental and theoretical studies, we demonstrate that the unique photoluminescence performance is caused by oxygen-vacancy-induced electron localization around the Bi^3+^ ions. This finding provides a new insight into the design of novel luminescence materials by changing the local electron structures of activator ions. The fabricated pc-WLED devices realize a high color rendering index (CRI = 95.1) and a low correlated color temperature (CCT = 5323 K), thereby indicating that LGO:Bi^3+^ is a superb red-emitting candidate in the field of solid-state lighting.

## Results

**Fig. 1:**
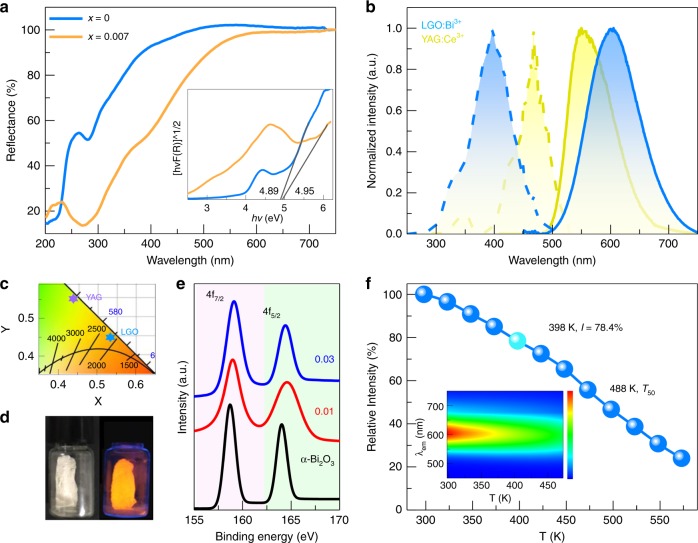
Photoluminescence properties and thermal stability analysis. **a** Diffuse reflectance (DR) spectra of La_4-*x*_GeO_8_:*x*Bi^3+^ (LGO:*x*Bi^3+^, *x* = 0, 0.007) samples; the inset presents the calculated optical bandgap values. **b** PLE and PL spectra of an LGO:0.007Bi^3+^ sample and commercially available yellow YAG:Ce^3+^ phosphor, which are monitored at the optimal wavelength. **c** The chromaticity coordinates of the representative LGO:0.007Bi^3+^ sample (*λ*_ex_ = 397 nm) and YAG:Ce^3+^ sample (λ_ex_ = 460 nm) in the CIE 1931 color space. **d** Photographs of LGO:0.007Bi^3+^ under natural light (left) and 365 nm n-UV light (right) radiation. **e** XPS spectra of LGO:*x*Bi^3+^ (*x* = 0.01, 0.03) samples and a standard α-Bi_2_O_3_ sample, which is used for comparison. **f** The variation in the integrated emission intensity (*λ*_ex_ = 397 nm) of LGO:0.007Bi^3+^ *vs* the heating temperature (298–573 K); the inset presents the corresponding temperature-dependent PL spectra

The optical performance of the novel orangish-red-emitting LGO:Bi^3+^ phosphor is evaluated in detail through diffuse reflectance (DR) spectra. The DR spectra of the LGO matrix show only one prominent band, which is centered at 280 nm (Fig. 1a). After Bi^3+^-doping (*x* = 0.007), in addition to the prominent band in the region of 232–321 nm, a shoulder band appears at ~400 nm. The former is mainly attributed to the matrix absorption of LGO and the ^1^S_0_→^1^P_1_ transition of Bi^3+^, while the latter originates from the ^1^S_0_→^3^P_1_ transition of Bi^3+,^^24^. The optical bandgap value can be obtained via linear extrapolation based on the DR spectra according to the following equations:^25^

1 $$[hv{{F}}({{R}})]^{ 1/2} = {{A}}(hv - {\mathrm{Eg}})$$

2 $${{F}}({{R}}) = (1 - {{R}})^{2}/2{{R}}$$

where *A* represents the absorption constant, *R* is the reflectance coefficient (%), *F*(*R*) is the absorption coefficient, Eg is the optical bandgap value, and *hv* represents the photon energy. The bandgap values for the LGO matrix and LGO:0.007Bi^3+^ are 4.89 and 4.95 eV, respectively (see the inset of Fig. 1a). These results demonstrate that the LGO matrix is a superior carrier for accommodating Bi^3+^ ions as inorganic luminescence materials.

Figure 1b presents the photoluminescence excitation (PLE) and photoluminescence emission (PL) spectra of the LGO:0.007Bi^3+^ sample and the best commercially available yellow YAG:Ce^3+^ phosphor at 298 K. The PLE spectrum of LGO:0.007Bi^3+^ shows a wide excitation band from 250–500 nm with a peak at 397 nm, thereby demonstrating that the as-prepared LGO: Bi^3+^ can be well excited by a n-UV LED chip. This result is consistent with the results from the DR spectra. In response to 397 nm n-UV light, LGO:0.007Bi^3+^ exhibits an unprecedented orangish-red emission from 500 to 750 nm with a maximum at 600 nm (fwhm = 103 nm). This emission can be attributed to the characteristic ^3^P_1_→^1^S_0_ transition of Bi^3+^. In the PL spectrum of LGO:Bi^3+^, an emission redshift of ~50 nm and a broader fwhm compared to those of YAG:Ce^3+^ (maximum at 550 nm and fwhm = 87.5 nm) are observed, thereby demonstrating that LGO:Bi^3+^ could cover more of the red component in PL spectra. Simultaneously, LGO:Bi^3+^ shows no reabsorption between the PLE and PL spectra, the IQE value can reach 88.3%, and EQE = 69%. The calculated Commission Internationale de l’Eclairage (CIE) diagrams for LGO:0.007Bi^3+^ (*λ*_ex_ = 397 nm) and YAG:Ce^3+^ (*λ*_ex_ = 460 nm) are located in the orangish-red region (0.536, 0.444) and yellow region (0.436, 0.560) (Fig. 1c), respectively. The luminescence photographs in Fig. 1d further demonstrate the bright orangish-red emission of LGO:Bi^3+^ upon 365 nm n-UV radiation. In addition, the detailed energy-level transition attribution at low-temperature (10 K) is consistent with the RT spectra (298 K) (Figure S1a and S1b), thereby demonstrating a stable Bi^3+^ luminescence. The Bi^3+^-doping optimization demonstrate that the Bi^3+^ critical concentration is *x* = 0.007 (Figure S1c-S1f). These results all demonstrate that LGO:Bi^3+^ could act as an orangish-red phosphor in n-UV-based pc-WLEDs and even has an advantage over the YAG:Ce^3+^ phosphor for presenting warm white light.

Since the valence of Bi has a critical influence on its luminescence properties, it is necessary to define its valence in the LGO matrix^26,27^. In Fig. 1e, the X-ray photoelectron spectroscopy (XPS) spectra of LGO:*x*Bi^3+^ (*x* = 0.01 and 0.03) samples and standard α-Bi_2_O_3_ powder are plotted. All samples show the typical Bi^3+^ peaks at ~159 and 164.6 eV, which are assigned to Bi 4*f*_7/2_ and Bi 4*f*_5/2_, respectively. In addition, when the LGO:Bi samples are treated under a N_2_/H_2_ (90%/10%) reducing atmosphere, no luminescence is observed. PL spectra that are treated in a different environment are shown in Figure S2. The results of this experiment demonstrate that the orangish-red emission in LGO originates from Bi^3+^ ions.

Typically, low thermal quenching behavior is necessary for phosphors to produce high-quality lighting in pc-WLED devices^28,29^. Fig. 1f exhibits temperature-dependent PL properties of LGO:0.007Bi^3+^. Although its emission intensity gradually declines as the temperature increases from 298 K to 573 K, it maintains 78.4% emission intensity at 398 K of the original intensity at 298 K. The quenching temperature, which is denoted as *T*_*50*_ (when emission intensity is half the original intensity), is 488 K. The quenching process is ascribed to the thermally excited nonradiative transition. Furthermore, the emission peak position and shape show almost no shift (inset of Fig. 1f) with increasing temperature in the region of 298–473 K, thereby demonstrating excellent color-stability. Furthermore, the temperature-dependent PL spectra and integrated intensity from 10 K to 300 K support the excellent temperature stability (Figure S3), thereby demonstrating that LGO:Bi^3+^ has high thermal stability for the practical application of pc-WLEDs.

**Fig. 2:**
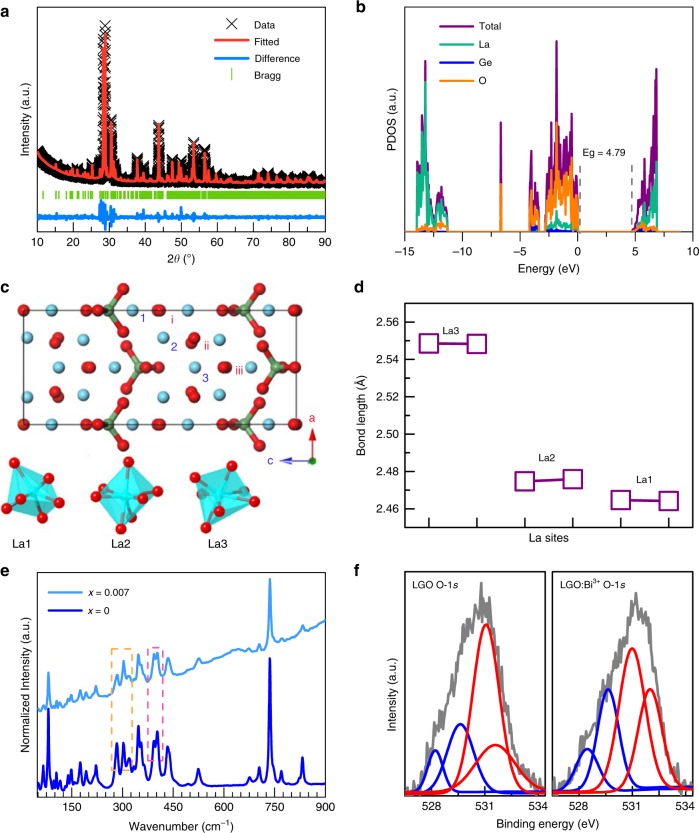
Structure characterizations of La_4_GeO_8_:Bi^3+^ phosphor and its defect formation. **a** X-ray powder diffraction (XRD) patterns of the LGO matrix with the measured data, fitted data, difference, and Bragg position, which are based on the Rietveld refinement. **b** The projected electronic density of states (PDOS) of the LGO matrix, which is obtained via DFT calculations. **c** The crystal structure of LGO from the *b*-axis direction and the polyhedral structure of La1–La3, where the green, red and blue spheres represent Ge, O, and La atoms, respectively. **d** The La–O bond length, which is obtained via DFT calculations. **e** Raman spectra of LGO:*x*Bi^3+^ (*x* = 0, 0.007). **f** XPS analysis of the O 1*s* orbital for the La_4_GeO_8_ matrix (LGO) and La_3.993_GeO_8_:0.007Bi^3+^ (LGO:Bi^3+^)

To explore the origin of the unique orangish-red emission of Bi^3+^ ions in LGO, the crystal structure configuration and composition of LGO:Bi^3+^ are investigated. The XRD patterns of LGO:*x*Bi^3+^ (*x* = 0.005‒0.030) could be indexed with the standard La_4_GeO_8_ (PDF No. 40–1185), thereby demonstrating the formation of a pure phase (Figure S4). Figure 2a and Figure S5a-S5c display the Rietveld refinement XRD patterns of LGO:*x*Bi^3+^ (*x* = 0‒0.03). All the as-prepared samples crystalize in orthorhombic unit cells with space group *P*1. The refined lattice parameters for LGO are *a* = 7.6642(8) Å, *b* = 5.8470(9) Å, *c* = 18.2897(4) Å, and *V* = 819.629(3) Å^3^ and the detailed structural information is listed in Table S1. Figure S5d plots the representative lattice parameter *c* and cell volume *V* as functions of the Bi^3+^ ion concentration *x*. The lattice parameters gradually decrease as *x* increases, which results from the smaller ionic radius of Bi^3+^ (1.03 Å, CN = 6; CN represents the coordination number) compared to La^3+^ (1.032 Å, CN = 6). Furthermore, the lattice parameters *a* and *b* gradually increase with *x*, thereby indicating that the La lattices may be locally distorted with the incorporation of Bi^3+^ (Figure S5e and S5f).

Density functional theory (DFT) calculations are used to further investigate the electron properties and structural configuration of the LGO:*x*Bi^3+^ (*x* = 0‒0.03) system. Figure 2b presents the projected electron density of states (PDOS) of the LGO matrix. According to the DFT calculations, the electrons of the conduction band are contributed by La atoms and O atoms, while the electrons of the valence band are mainly contributed by O atoms. The calculated bandgap (Eg) value (4.79 eV) is close to the experimental value (4.89 eV). The crystal structure that is calculated via the DFT method is depicted schematically in Fig. 2c. Along the *b*-axis direction, LGO exhibits a highly symmetric structure, which consists of three types of [LaO_*n*_] polyhedra (*n* = 6, 7) and two types of [GeO_4_] tetrahedra. The six crystallographic La sites can be sorted into three categories: La1–La3. The La1 site is connected with six O atoms to form a distorted octahedron, while the La2 and La3 sites are coordinated with seven O atoms to form decahedra. The O atoms in LGO can also be divided into three types: Oi-Oiii. The structural information that is obtained via DFT calculations well agrees with the Rietveld refinement results. The calculated average La–O bond lengths are summarized in Fig. 2d and Table S2, which are 2.4642 Å for La1–O, 2.4759 Å for La2–O, and 2.5486 Å for La3–O.

The composition of LGO:*x*Bi^3+^ (*x* = 0, 0.007, 0.030) is determined from FT-IR spectra (Figure S6) and Raman spectra (Fig. 2e). In the FT-IR spectra, the stronger peak at 683 cm^−1^ and weaker peak at 709 cm^−1^ are typically antisymmetric vibrations of GeO_4_ tetrahedra^30^, while the peak at 502 cm^−1^ corresponds to characteristic stretching vibrations of the La–O bond. Due to the scant Bi^3+^-doping concentration, the incorporation of Bi^3+^ does not influence the structures of the as-prepared samples. Raman spectra also support the phase composition of LGO. The relative intensities of the peaks at 284.4 cm^−1^ and 303.6 cm^−1^ differ substantially between the *x* = 0 and *x* = 0.007 samples (marked by an orange dashed rectangle). The peak splitting degree at ~400 cm^−1^ also differs between the two samples (marked by a pink dashed rectangle). These variations in the Raman spectra demonstrate that the local lattice coordination environment in the LGO matrix changes slightly with Bi^3+^ ion incorporation and may result in the formation of lattice defects. XPS analysis is an effective method for elucidating the presence of vacancies. With the doping of Bi^3+^ into the LGO matrix, the 3*d* orbital of La and the Ge binding energy remain unchanged (Figure S8). The O 1*s* orbital could be fitted by four Gaussian peaks that are centered at ~528.2, ~529.6, ~531, and ~532 eV in both the LGO and LGO:Bi^3+^ samples (Fig. 2f). Accordingly, the high-energy component (red lines) is mainly attributed to loosely bound oxygen, whereas the two low-energy peaks (blue lines) are ascribed to the presence of oxygen vacancies^31^. The low-energy peaks increase in intensity with Bi^3+^ incorporation, thereby indicating the generation of oxygen vacancies in the LGO:Bi^3+^ sample.

**Fig. 3:**
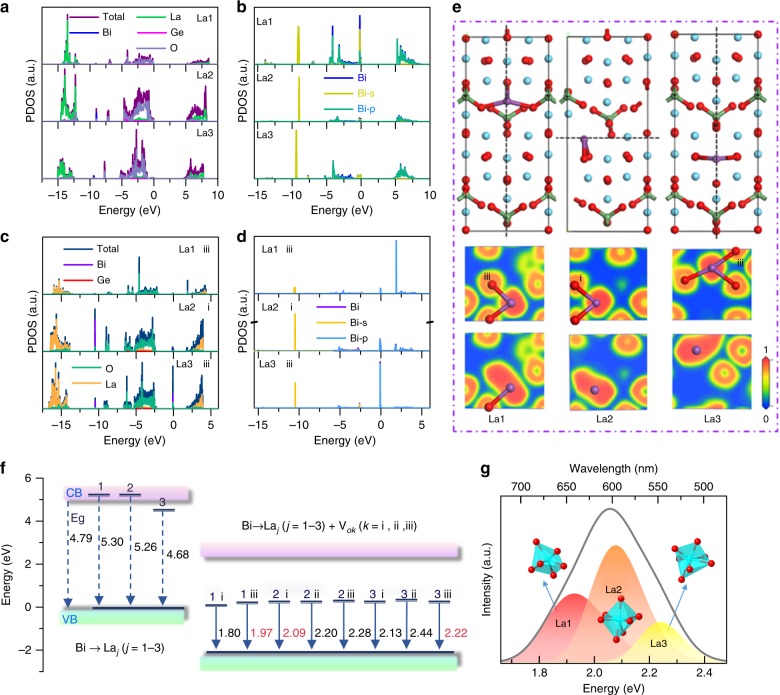
DFT calculation and ELF maps for La_4_GeO_8_:Bi^3+^ phosphor. **a**–**d** Projected electronic densities of states (PDOSs) for three types of La sites, which are obtained via DFT calculations, where **a** and **b** are without oxygen vacancies and **c** and **d** are with oxygen vacancies. **e** Crystal structure of Bi^3+^-doping into various La^3+^ sites and the electron localization function (ELF) maps without (up) and with (down) oxygen vacancies in three La sites, where the purple, blue, green and red spheres represent Bi, La, Ge, and O atoms, respectively. **f** A schematic diagram of the transition energy levels of Bi replacing La without and with oxygen vacancies (i–iii) between the valence band and the conduction band. **g** The Gaussian fitting peak of the representative LGO:0.007Bi^3+^ sample

Enlightened by the crystal structure and lattice environment analysis, we posit that Bi^3+^ ions randomly occupy all La sites. DFT calculations are used to investigate the local electron structure variation around the Bi^3+^ ions and coordinated O atoms. The calculated total and partial electron densities of state of the La1–La3 sites in LGO:0.007Bi^3+^ are displayed in Fig. 3a and b. The locations and contributions of the La, Ge, and O atoms in LGO:Bi^3+^ are coincident with those in the LGO matrix. Surprisingly, no electron transition energy levels of the Bi^3+^ ions are observed between the conduction band and valence band. In Fig. 3b, the extracted PDOSs for the total and *s* and *p* energy levels of the Bi atoms support the absence of an electron transition energy level between the conduction band and the valence band of LGO. The calculated results demonstrate that there is no luminescence when doping Bi^3+^ ions in the LGO matrix, which contradicts the experimental results.

According to the previous Raman results, slight changes occur with Bi^3+^ ion doping into the LGO matrix; hence, defects or distortions in the local lattice coordination environment are expected. The calculated La1 site distortion degree is increased from 0.2832 for the LGO matrix to 0.2943 for LGO:0.007Bi^3+^; the distortion degree is calculated as $D = \frac{1}{n}\mathop {\sum}\limits_{i = 1}^n {\frac{{\left| {d_i - d_{{\mathrm{av}}}} \right|}}{{d_{{\mathrm{av}}}}}}$^32^, where *D* represents the lattice distortion, *d*_*i*_ is the distance from Ba to the *i*th coordinating O atom, *d*_av_ is the average Ba–O distance, and *n* is the coordination number. Among the various defects, oxygen vacancies are easily generated during the annealing process of Bi-activated inorganic phosphors^18,23^. Then, we calculate the electron structures of LGO:Bi^3+^ with three types oxygen-vacancy defects at the La1–La3 sites; the results are exhibited in Fig. 3c, d and Figure S7. The whole electron distribution shifts in the low-energy direction by ~2.5 eV. The electron transition energy levels of the Bi 6*p* orbital appear between the conduction band and the valence band of the LGO matrix. Thus, we conjecture that the existence of oxygen-vacancy defects is extremely important for the generation of orangish-red emission in LGO:Bi^3+^.

To further investigate the influence of oxygen vacancies on the Bi^3+^ luminescence behavior in the LGO matrix, the electron localization function (ELF) maps around the Bi atoms at the La1–La3 sites (Fig. 3e) without and with oxygen vacancies are analyzed. When Bi atoms substitute La sites, many electrons will localize around the Bi atoms and fewer electrons will be concentrated on coordinated O atoms. Comparing the ELF maps without (up) and with (down) oxygen vacancies at the La1–La3 sites, the electron localization area around the Bi atom enlarges as an adjacent oxygen vacancy appears. Accordingly, the unique orangish-red emission of LGO:Bi^3+^ is mainly ascribed to the electron localization around Bi atoms in the presence of oxygen vacancies. The proposed electron transition, which is based on DFT calculations, is summarized schematically in Fig. 3f. When Bi^3+^ ions occupy La^3+^ ions without generating oxygen vacancies, the Bi 6*p* energy level embeds into the conduction band, while the 6*s* energy level is close to the valence band. The separation energy between the 6*s* and 6*p* energy levels is almost equal to the bandgap value; thus, there are no electron transitions on Bi^3+^ ions for generating luminescence. In the presence of an oxygen vacancy, the Bi 6*p* excited-state energy level appears between the conduction band and the valence band and the 6*s* energy level mainly embeds into the valence band maximum. The separation energies of the 6*s* and 6*p* energy levels with various oxygen vacancies at the La1–La3 sites are in the range of 1.80–2.44 eV and the statistical separation energies in ascending order are La1 < La2 < La3. These results demonstrate that Bi^3+^ can randomly occupy the La1, La2, and La3 sites in the LGO matrix and, theoretically, emits yellow to deep-red light in the presence of oxygen vacancies at the La1‒La3 sites. Our experimental results well agree with this theoretical prediction. The Gaussian fitting PL spectra (Fig. 3g) and lifetime values (Figure S9, 1.39, 1.34, and 1.28 μs for the La1–La3 sites, respectively) for LGO:0.007Bi^3+^ support the existence of three luminescence centers with peak positions at 1.93 eV (650 nm), 2.08 eV (600 nm), and 2.24 eV (550 nm). The crystal splitting field energy (Dq) is expressed as follows:^33,34^

3 $$D_{\mathrm{q}} = \frac{{Ze^2r^4}}{{6R^5}}$$

where *Z* is the charge of the anion, *e* is the charge of one electron, *r* is the radius of the *d* wave function, and *R* is the average La–O bond length. As the bond length decreases, the crystal field splitting increases. On the basis of Fig. 2d and Table S2, the deep-red (650 nm), orange (600 nm), and yellow (550 nm) luminescence centers are assigned to the occupation of Bi^3+^ at the La1, La2, and La3 sites, respectively. The oxygen-vacancy-induced electron localization around the Bi atoms is the crucial factor for generating orangish-red emission in the LGO:Bi^3+^ phosphor.

**Fig. 4:**
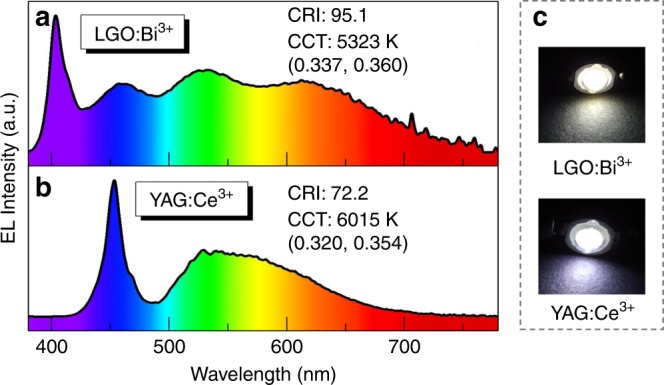
WLEDs applications for La_4_GeO_8_:Bi^3+^ phosphor. EL spectra of pc-WLEDs that were fabricated from **a** an orangish-red LGO:0.007Bi^3+^ sample, blue BAM:Eu^2+^ phosphor and green Ba_3_Si_6_O_12_N_2_:Eu^2+^ phosphor with a 400 nm LED chip and **b** yellow YAG:Ce^3+^ phosphor with a 460 nm LED chip. **c** Photographs of the pc-WLED devices that were fabricated from LGO:Bi^3+^ and YAG:Ce^3+^ phosphors

To evaluate the practical application of LGO:Bi^3+^ phosphor in a pc-WLED device, we fabricated a pc-WLED device by using LGO:0.007Bi^3+^, commercial green Ba_3_Si_6_O_12_N_2_:Eu^2+^ phosphor, blue BAM:Eu^2+^ phosphor, and a 400 nm n-UV LED chip. For comparison, the pc-WLED is fabricated similarly by using commercial YAG:Ce^3+^ phosphor with a 460 nm LED chip. The electroluminescence (EL) spectra are presented in Fig. 4a, b, which are consistent with their corresponding PL spectra. Under the optimal LED chip excitation, LGO:0.007Bi^3+^ covers more of the red component in the visible light region than YAG:Ce^3+^. With a 3 V, 20 mA current driving, the CCT (correlated color temperature) and CRI (color rendering index) of the as-fabricated pc-WLEDs for LGO:0.007Bi^3+^ are 5323 K and 95.2, respectively, which are superior compared to the commercial YAG:Ce^3+^ phosphor (6015 K and 72.2). The luminous efficiency of the as-fabricated pc-WLEDs for LGO:0.007Bi^3+^ is 6.4 lm/W, which should be optimized via proper process treatment. Moreover, the CIE color coordinates of the former (0.337, 0.360) correspond to a more suitable white-emitting position than those of the latter (0.320, 0.354). The fabricated pc-WLED of LGO:Bi^3+^ exhibits much warmer white light than that of YAG:Ce^3+^ (Fig. 4c). These results demonstrate that the developed LGO:Bi^3+^ is a promising orangish-red phosphor material for application in n-UV-based warm pc-WLEDs lighting.

## Discussion

In summary, we have successfully exploited an orangish-red-emitting LGO:Bi^3+^ phosphor with the emission peak locating at 600 nm (*λ*_ex_ = 397 nm) and the fwhm = 103 nm. Its IQE could reach 88.3%, EQE = 69%. Rietveld refinement confirms that the as-prepared samples crystalize in orthorhombic unit cells with space group *P*1, and its lattice parameters are *a* = 7.6642(8) Å, *b* = 5.8470(9) Å, *c* = 18.2897(4) Å, and *V* = 819.629(3) Å^3^. The XRD spectra and DFT calculation results demonstrate that Bi^3+^ ions randomly occupy La1–La3 sites in LGO, which will emit deep-red (1.93 eV), orange (2.08 eV), and yellow (2.24 eV) light when substituting at La1, La2, and La3 sites, respectively. Interestingly, the unique orangish-red luminescence behavior should be ascribed to the electron localization surrounding Bi^3+^ ions with the presence of adjacent O vacant defects through ELF maps analysis. The fabricated n-UV-based pc-WLED with a hybrid of LGO:Bi^3+^ phosphor, blue BAM:Eu^2+^ and green Ba_3_Si_6_O_12_N_2_:Eu^2+^ phosphor achieves high CRI (95.2) and low CCT (5323 K), indicating a superb candidate in lighting field. The concept of focusing on the electron localization properties surrounding activators ions offers a new perspective and insight for exploring unique luminescence behavior in inorganic luminescence materials.

## Materials and methods

### Materials synthesis

Samples of La_4-*x*_GeO_8_:*x*Bi^3+^ (*x* = 0‒0.03), which is denoted as LGO:*x*Bi^3+^, were prepared via a conventional high-temperature solid-state approach. The raw materials of La_2_O_3_ (99.99%), GeO_2_ (99.999%) and Bi_2_O_3_ (99.99%) were all purchased from Aladdin. First, stoichiometric raw materials were weighed, put into agate, and ground together for 1 h. The obtained mixtures were transferred into corundum crucibles and annealed in a horizontal tube furnace at 1100‒1300 °C for 4 h in air. After naturally cooling to room temperature, the annealed samples were ground again. In addition, La_4-*x*_GeO_8_:*x*Bi^3+^ (*x* = 0‒0.03) samples were prepared under an N_2_/H_2_ (90%/10%) reduced atmosphere with the same other conditions for comparison.

### LED fabrication

pc-WLED devices were fabricated by using the as-prepared orangish-red LGO:0.007Bi^3+^ phosphor, blue BAM:Eu^2+^ phosphor, green Ba_3_Si_6_O_12_N_2_:Eu^2+^ phosphor, and a 400 nm LED chip. In a typical fabrication process, the LGO:0.007Bi^3+,^ BAM:Eu^2+^ and Ba_3_Si_6_O_12_N_2_:Eu^2+^ phosphors were evenly blended with silicone resins A and B (A:B = 1:1) in the agate mortar and the resulting mixture was coated on a 400 nm LED chip. The packaged devices were cured in an oven at 120℃ for 12 h to form the resulting pc-WLED devices. The commercially available yellow YAG:Ce^3+^ phosphor and a 460 nm LED chip were also fabricated to pc-WLED via the same method for comparison.

### Characterization

Powder X-ray diffraction (XRD) were collected on a D8 Focus diffractometer with Ni-filtered Cu-Kα (2θ = 5°–20°, λ = 1.540598 Å). Rietveld refinements were performed with General Structure Analysis System (GSAS) software based on XRD data. Photoluminescence excitation (PLE) and photoluminescence emission (PL) spectra were collected by a fluorescence spectrometer (Fluoromax-4P, Horiba Jobin Yvon, New Jersey, U.S.A.) whose excitation source is a 450 W xenon lamp. Diffuse reflectance (DR) spectra were collected with using a UV–vis-NIR spectrophotometer (Hitachi U-4100). Photoluminescence decay curves were obtained with using a Lecroy Wave Runner 6100 Digital Osilloscope (1 GHz) (Contimuum Sunlite OPO), the excitation was a tunable laser (pulse width = 4 ns and gate = 50 ns). Fourier-transform infrared spectra (FT-IR) were performed on spectrophotometer (Bruker, Vertex Perkin–Elmer 580BIR) by using KBr pellet technique. Raman spectra were obtained on Raman spectrometer (JYT6400) using a 512 nm laser. The photoluminescence quantum yield (QY) was obtained on an absolute PL quantum yield measurement system (Hamamatsu photonics K.K., C9920-02 Japan). The electroluminescence performances of pc-WLED devices were measured analyzer system (tarspec SSP6612.) by using an integrating sphere. All the above measurements were performed at room temperature. Temperature-dependent PL spectra (10–300 K and 298–573 K) were recorded on a fluorescence spectrophotometer (Edinburgh Instruments FLSP-920) with a temperature controller.

### Computational methods

The first-principle density functional theory (DFT) calculations were performed with the Vienna ab initio simulation package (VASP) code. The electron–ion interaction was treated with the projector augmented wave (PAW) method. La (5*s*^2^5*p*^6^5*d*^1^6*s*^2^), Ge (4*s*^2^4*p*^2^), O (2*s*^2^2*p*^4^) and Bi (6*s*^2^6*p*^3^) electrons were treated as the valence electrons. The exchange and correlation functional were described via the Perdew–Burke–Ernzerhof (PBE) generalized gradient approximation. To investigate the electronic properties and obtain an accurate description of the density of states, we employed the Heyd–Scuseria–Ernzerhof (HSE06) method. A plane-wave cutoff energy of 400 eV was applied in our calculations and 4×4×2 Monkhorst–Pack *k* grids were used during the optimization. The iterative process was considered to have converged when the force on the atom was less than 0.01 eV Å^−1^ and the energy change was <10^–5^ eV per atom.

## Supplementary information

supplemental materials
