# Supplementary material for: New strategy for designing orangish-red-emitting phosphor via oxygen-vacancy-induced electronic localization
Source: Light Sci Appl. 2019 Jan 30;8:15. doi: 10.1038/s41377-019-0126-1 (PMC6351663; doi:10.1038/s41377-019-0126-1)
Supplement: Supplementary file 1 — supplemental materials [file 41377_2019_126_MOESM1_ESM.docx]

New Strategy for Designing Orangish-Red Emitting Phosphor via Oxygen-Vacancy-Induced Electronic Localization

Yi Wei,^1^ Gongcheng Xing,^1^ Kang Liu,^2, 3^ Guogang Li,^1,^ * Peipei Dang,^2^ Sisi Liang,^2^ Min Liu,^3,4,^* Ziyong Cheng,^2^ Dayong Jin,^5^ and Jun Lin^2, 6,^ *

*^1^* Engineering Research Center of Nano-Geomaterials of Ministry of Education, Faculty of Materials Science and Chemistry, China University of Geosciences, 388 Lumo Road, Wuhan 430074, P. R. China. Email: ggli@cug.edu.cn

*^2^* State Key Laboratory of Rare Earth Resource Utilization, Changchun Institute of Applied Chemistry, Chinese Academy of Sciences, Changchun 130022, P. R. China*.* Email: jlin@ciac.ac.cn

*^3^* Hunan Key Laboratory for Super-microstructure and Ultrafast Process, School of Physics and Electronics, Central South University, Changsha, Hunan 410083, P. R. China. Email: minliu@csu.edu.cn

*^4^* State Key Laboratory of Powder Metallurgy, Central South University, 932 South Lushan Road, Changsha, Hunan 410083, P. R. China

*^5^* Institute for Biomedical Materials and Devices (IBMD), Faculty of Science, University of Technology Sydney, Sydney, NSW, Australia

*^6^* School of Applied Physics and Materials, Wuyi University, Jiangmen, Guangdong, 529020, P. R. China

These authors contributed equally: Yi Wei, Gongcheng Xing, Kang Liu

**Figure S1.** The PLE (monitored at 610 nm) and PL spectra (monitored at 397 nm) of LGO:0.007Bi^3+^ sample at the temperature of (a) 10 K and (b) 298 K. (c) The PLE spectra of LGO:*x*Bi^3+^ (*x* = 0.005-0.030) samples monitored at 600 nm. (d) The PL spectra of LGO:*x*Bi^3+^ (*x* = 0.005-0.030) samples monitored at 397 nm. (e) The fwhm and peak position as functions of doping Bi^3+^ ions concentration (*x*). (f) The integrated intensity as function of the doping Bi^3+^ ions concentration (*x*).

The PLE and PL spectra of LGO:0.007Bi^3+^ sample measured at different temperature (10 K and 298 K) are presented in Figure S1a and S1b. The PLE spectra shows broad absorption from 250-500 nm with the peak position locating at 397 nm, owing to ^1^S_0_ → ^3^P_1_ electron translation of Bi^3+^ ions, while a shoulder peak located around 329 nm is due to the ^1^S_0_ → ^1^P_1_ transition of Bi^3+^ ions. It could be observed that the when measuring at 10 K, an additional shoulder peak at 292 nm occurs. We infer that it belongs to the La_4_GeO_8_ matrix absorption band. The PL spectra of LGO:0.007Bi^3+^ displays broad orange-red emission band with the peak position at 600 nm, which is ascribed to ^3^P_1_ → ^1^S_0_ electron transition of Bi^3+^ ions. Figure S1c and S1d show the PLE and normalized PL spectra of LGO:*x*Bi^3+^ (*x* = 0.005-0.030) samples, respectively. It could be observed that the profile and shape maintain unchanged as Bi^3+^ content increases. The fwhms and emission peak position are summarized in Figure S1e, it is noted that the fwhms basically remain unchanged in the region of 103-106 nm. Meanwhile, the emission peak position does not shift as Bi^3+^ doping content increases. In addition, the integrated emission intensity reaches maximum at *x* = 0.007 (Figure S1f), subsequently decreases when Bi^3+^ concentration is beyond 0.007 due to concentration quenching.

**Figure S2.** The PL spectra (monitored at 397 nm) of LGO:0.007Bi^3+^ sample measured at air and N_2_/H_2_ gas.

**Figure S3.** (a) The temperature-dependent PL spectra of LGO:0.007Bi^3+^ sample from 10 K to 300 K with an interval of 20 K. (b) The normalized integrated intensity as function of temperature from 10 K to 300 K in LGO:0.007Bi^3+^ sample.

Under 400 nm n-UV light excitation, Figure S2a presents the temperature-dependent PL spectra in the region of 10 K-300 K, it could be observed that the emission intensity gradually decreases as the temperature increases. In addition, the peak position appears slightly blue-shift, which could be attributed to the thermal excited non-radiation transition process. the integrated emission intensity is plotted in Figure S2b, indicating that integrated intensity could maintain beyond 80% at 300 K of the initial intensity at 10 K. This result clarifies the thermal quenching of LGO:Bi^3+^ is acceptable from low temperature to RT.

**Figure S4.** The XRD patterns of LGO:*x*Bi^3+^ (*x* = 0.005-0.030) samples and the standard La_4_GeO_8_ (PDF No. 40-1185) at room temperature. The black rhombus (◆) represents the slight impurity of La_2_GeO_5_.

**Figure S5.** XRD patterns of the representative LGO:*x*Bi^3+^ (*x* = 0-0.030) samples with the measured data and fitted profile based on the Rietveld refinement, where (a) *x* = 0.005; (b) *x* = 0.007; (c) *x* = 0.030. (d) The calculated lattice parameters *c* and cell volume *V* as a function of Bi^3+^ concentration (*x*). (e, f) The calculated lattice parameters *a* and *b* as a function of Bi^3+^ concentration (*x*).

**Figure S6.** FT-IR spectra of LGO:*x*Bi^3+^ (*x* = 0, 0.007).

**Figure S7.** Projected electronic density of states (PDOSs) of LGO:Bi^3+^ samples. Bi^3+^ ions incorporate into different La sites with diverse O sites, respectively. (a, b) Bi^3+^ ions incorporate into La1 site. (c, d) Bi^3+^ ions incorporate into La2 site. (e, f) Bi^3+^ ions incorporate into La3 site.

**Figure S8.** XPS analysis of La-3*d* orbital and Ge-3*d* orbital for La_4_GeO_8_ matrix (LGO) and La_3.993_GeO_8_:0.007Bi^3+^ (LGO:Bi^3+^).

**Figure S9.** Photoluminescence decay curves for three kinds of La sites in LGO:0.007Bi^3+^ sample measured at λ_ex_ = 400 nm. The inset is the decay lifetime values at three La sites.

In Figure S8, the luminescence lifetime decay curves for three kinds of La sites in LGO:0.007Bi^3+^ sample are monitored at λ_ex_ = 400 nm and λ_em_ = 560 nm, λ_em_ = 600 nm, λ_em_ = 656 nm, respectively. The decay curves could be successfully fitted using monoexponential equation:

$\mathrm{I}(\tau)=\mathrm{I}_{0}+A*\exp(-\tau/\mathrm{t})$ (1)

where I(τ) and I_0_ are the luminescence intensities at time τ, A is fitting constant; and t represents the decay time of the exponential components. The fitted decay lifetime values are 1.28 μs, 1.33 μs, 1.39 μs, which agrees well with the typical Bi^3+^ lifetime value.

**Table S1.** Crystallographic Parameters Obtained from XRD Rietveld Refinement for LGO:*x*Bi^3+^ (*x* = 0-0.030).

| Samples | Space group | Crystallographic parameters | | | | Reliability factors | | |
| --- | --- | --- | --- | --- | --- | --- | --- | --- |
|  |  | *a* (Å) | *b* (Å) | *c* (Å) | *V* (Å^3^) | *R_p_* | *R_wp_* | *χ*^2^ |
| *x* = 0 | Orthorhombic  *P*1 | 7.6642(8) | 5.8470(9) | 18.2897(4) | 819.629(3) | 7.27% | 10.95% | 6.247 |
| *x* = 0.005 |  | 7.6642(8) | 5.8471(2) | 18.2819(4) | 819.287(3) | 6.90% | 10.29% | 6.071 |
| *x* = 0.007 |  | 7.6638(9) | 5.8469(0) | 18.2806(4) | 819.151(3) | 7.62% | 11.35% | 6.827 |
| *x* = 0.030 |  | 7.6667(2) | 5.8493(5) | 18.2625(4) | 818.989(3) | 6.78% | 10.16% | 4.573 |

**Table S2**. The La–O Bond Length of Six La Sites Polyhedrons in La_4_GeO_8_ Matrix.

| Bond | Length (Å) | Bond | Length (Å) | Bond | Length (Å) | Bond | Length (Å) | Bond | Length (Å) | Bond | Length (Å) |
| --- | --- | --- | --- | --- | --- | --- | --- | --- | --- | --- | --- |
| La3–O | 2.318 | La3’–O | 2.318 | La1–O | 2.347 | La2–O | 2.428 | La2’–O | 2.428 | La1’–O | 2.347 |
| La3–O | 2.324 | La3’–O | 2.324 | La1–O | 2.388 | La2–O | 2.428 | La2’–O | 2.428 | La1’–O | 2.388 |
| La3–O | 2.553 | La3’–O | 2.553 | La1–O | 2.400 | La2–O | 2.431 | La2’–O | 2.431 | La1’–O | 2.400 |
| La3–O | 2.553 | La3’–O | 2.553 | La1–O | 2.406 | La2–O | 2.498 | La2’–O | 2.499 | La1’–O | 2.406 |
| La3–O | 2.652 | La3’–O | 2.652 | La1–O | 2.575 | La2–O | 2.514 | La2’–O | 2.514 | La1’–O | 2.575 |
| La3–O | 2.72 | La3’–O | 2.72 | La1–O | 2.669 | La2–O | 2.516 | La2’–O | 2.516 | La1’–O | 2.669 |
| La3–O | 2.72 | La3’–O | 2.72 | — | — | La2–O | 2.516 | La2’–O | 2.516 | — | — |
| Average | 2.5486 | Average | 2.5486 | Average | 2.4642 | Average | 2.4759 | Average | 2.4759 | Average | 2.4642 |
